# Assessment of Panorama Photogrammetry as a Tool for Long-Range Deformation Monitoring

**DOI:** 10.3390/s24113298

**Published:** 2024-05-22

**Authors:** Peyman Javadi, Luis García-Asenjo, Raquel Luján, José Luis Lerma

**Affiliations:** Department of Cartographic Engineering, Geodesy and Photogrammetry, Universitat Politècnica de València, Camino de Vera s/n, 46022 Valencia, Spain; lugarcia@cgf.upv.es (L.G.-A.); ralugar@cgf.upv.es (R.L.); jllerma@cgf.upv.es (J.L.L.)

**Keywords:** panorama photogrammetry, terrestrial laser scanning (TLS), deformation monitoring, panoramic image, long distances, geodetic network, photomonitoring

## Abstract

This study investigates panorama photogrammetry (PPh) as a potential method to collect massive 3D information for long-range deformation monitoring. Particularly, this study focuses on areas with measuring restrictions, i.e., inaccessible objects and distances above 0.6 km. Under these particular conditions, geodetic techniques based on Electromagnetic Distance Meters (EDMs) or Total Stations (TSs) can provide coordinates with a precision better than 1 cm, but only for a limited number of discrete points. For mass capture, Terrestrial Laser Scanning (TLS) is normally the preferred solution, but long-range instruments are expensive, and drawbacks such as weak return signals and non-automatic target recognition appear. As an alternative, PPh is investigated in the well-controlled area of La Muela in Cortes de Pallas, where images are automatically captured from geodetic pillars using a GigaPan device, processed, and then rigorously compared to TLS point clouds. The results obtained after integrating both techniques into a high-accuracy geodetic reference frame show that PPh and TLS provide similar precision to within approximately 4 cm in the range of 0.6–1.0 km. Therefore, considering cost-effectiveness and ease of use, the proposed method can be considered a low-cost alternative to TLS for long-range deformation monitoring.

## 1. Introduction

Traditionally, deformation monitoring based on geodetic surveying has relied on Total Stations (TSs), Electro Distance Meters (EDMs), or Global Navigation Satellite Systems (GNSSs) [1,2,3,4]. Even at distances above 0.6 km, these techniques are able to provide consistent 3D coordinates with an accuracy of some millimetres in a well-defined terrestrial reference frame [5,6,7]. However, since they require careful operation and proper addressing of possible sources of error such as atmospheric refraction or instrument calibration, they tend to be time-consuming and can only be rigorously applied to a limited number of accessible points. This is acceptable for providing accurate reference frames and control points (CPs), but it is a serious limitation when it comes to monitoring vast areas, inaccessible terrains, or areas with safety concerns [1].

Current deformation monitoring projects requiring vast areas of monitoring normally include remote sensing techniques such as Terrestrial Laser Scanning (TLS) or Terrestrial Photogrammetry (TPh) [8]. Particularly, in the present case study conducted in Cortes de Pallas (Spain), which is described in Section 2, the detection of possible displacements of a few centimetres with the required level of significance in a short period, e.g., several years, requires the joint use of diverse geomatics techniques that have to be rigorously integrated to serve the following three components: monitoring a precise geodetic reference frame, reliable determination of possible displacements of a discrete number of critical points to detect movements of huge boulders or malfunctioning of the anchoring systems, and overall monitoring of the whole area of interest by using techniques able to collect efficiently massive information such as TLS or TPh [9,10].

The two first components were successfully approached by using a sub-millimetric EDM-based methodology able to provide very accurate 3D coordinates periodically, i.e., an accuracy better than 1 mm and 3 mm in the horizontal and vertical components, respectively [5,6]. However, the third component, originally carried out by using both TLS and TPh, showed that the use of these remote sensing techniques under a traditional approach may entail recurrent problems when they are applied to deformation monitoring in large areas with strong orography and physical obstructions [8,11]. The problems that are usually found can be classified into three types as follows: (1) difficulties in setting up stations that are well distributed near the area of interest, (2) difficulties in either the TLS registration process or the absolute orientation of the TPh models because of the lack of CPs in the target area, and (3) difficulties in obtaining full coverage leading to data gaps in the point clouds [9,12,13].

Particularly, problems concerning types (1) and (2) were found to be critical because proper integration of all the measuring campaigns into the same reference frame, which is realized in this case by a well-controlled geodetic network, is paramount to rigorous deformation monitoring analysis [5,10,14,15,16,17,18]. From our experience, the difficulties found are different for TLS and TPh. In the case of TLS, which is normally the first option for its easiness of operation, the following drawbacks can be pointed out: long-range TLS instruments are expensive and time-consuming, i.e., one hour per station is required for a dense point cloud, and weak return signals over long ranges are a recurrent issue, there is no possibility of automatic target recognition, and large targets are required. As a consequence, the registration process cannot be carried out with the required precision, and point clouds are sub-optimally integrated into the existing geodetic reference frame. On the other hand, TPh is a low-cost solution that can provide dense information and detect smaller targets, but it normally involves drawbacks such as the requirement of many stations, which entails careful planning, and the need for as many CPs as possible that are well distributed on the scene of interest, which sometimes are inaccessible (as in the current case study).

On the whole, TPh and TLS co-registration problems are mostly caused by a lack of proper CPs. Normally, they do not take advantage of the fact that the instrument’s position can be accurately known in many cases, and therefore require a large number of accurate and well-distributed CPs. However, in long-range deformation monitoring, the critical area tends to be neither stable nor accessible, which necessarily means that the number of available CPs is largely limited with the consequent impact on the absolute (external) orientation of the point clouds obtained. This assertion can be easily demonstrated by checking the position of TLS instruments or the TPh perspective centre once the registration process has been carried out.

Therefore, we decided to investigate an alternative approach based on PPh to cope with the aforementioned problems with both TLS and TPh. Assuming the co-registration problems are solved, the use of PPh for long-range deformation monitoring can be an innovative and cost-effective solution comparable to TLS with additional advantages, thus bridging the gap between traditional monitoring techniques and the demand for increased coverage and accuracy.

Generally, PPh, sometimes referred to as wide format photography, is a unique method that combines several photographs taken from the same camera position to create a single image with a field of view that is larger than or comparable to that of the human eye. But when this photography technique is combined with the principles of photogrammetry, it provides the possibility of producing point clouds, 3D models, and other products that can be measured [14,19]. Although this technique has been mainly used in geosciences as a qualitative way to represent and interpret high-resolution scenarios [20,21], recent works, and this study in particular, have examined the potential of this method for quantitative analysis in deformation monitoring [22,23]. This study introduces a special method for performing absolute orientation, which is different from the traditional method that is commonly used. Shooting is performed with the panorama technique, but image processing proceeds in a frame-based way. In previous studies [14,24], it was concluded that capturing a 360° view is not always necessary when only a portion of the real-world scene is of interest. Instead, limiting PPh to a specific area can enhance speed, accuracy, and quality. Furthermore, when images are captured using digital cameras in frame format, there is no need for image stitching, and the processing can be performed in a frame-based manner. The proposed method is based on the automatic collection of images using a GigaPan device from stable stations belonging to a well-controlled geodetic reference frame. Those stable stations, i.e., permanent geodetic pillars in the ongoing case study, are alternatively used as Gigapan stations or as CPs by setting up dedicated target spheres. This measuring strategy greatly strengthens the geometry even though the number of available CPs located in the critical area is limited, and it also ensures that all the images are properly integrated into a unique reference frame.

In summary, this work demonstrates that the joint use of panoramic images taken from pillars of a geodetic network used as a reference frame can yield similar accuracy as TLS, with additional advantages, thus meeting the requirements for deformation monitoring. This general objective is examined on two levels. First, the method’s ability to capture subtle displacements and deformations with a level of accuracy comparable to established techniques such as TLS. Second, to comprehensively understand the sources of error and challenges inherent to PPh when applied to deformation monitoring conducted in challenging orographic conditions [8,18].

The remainder of this paper is structured as follows. The Section 2 introduces the selected case study in Cortes de Pallás in the context of this investigation. The Section 3 describes the experimental setup, the methods and instruments used, and the processing procedures. In Section 4, the obtained results are discussed. Finally, Section 5 summarizes the gained knowledge, highlights the main limitations of the presented approach, and provides an outlook for future work.

## 2. Case Study

As a case study for the assessment of PPh, we selected the area known as La Muela in Cortes de Pallás (Spain), where the Diputació de València and the Universitat Politècnica de València have collaborated on a long-term deformation monitoring project since 2017. The reasons that explain why this area perfectly suits the purpose of this study are as follows: (1) the complex orography that encompasses serious geometric limitations and (2) the existence of a well-controlled permanent geodetic network established in 2017. The latter serves to properly integrate all the measuring campaigns into the same reference frame, which is paramount to rigorous deformation monitoring analysis. Both aspects of the case study are explained below.

**Figure 1:**
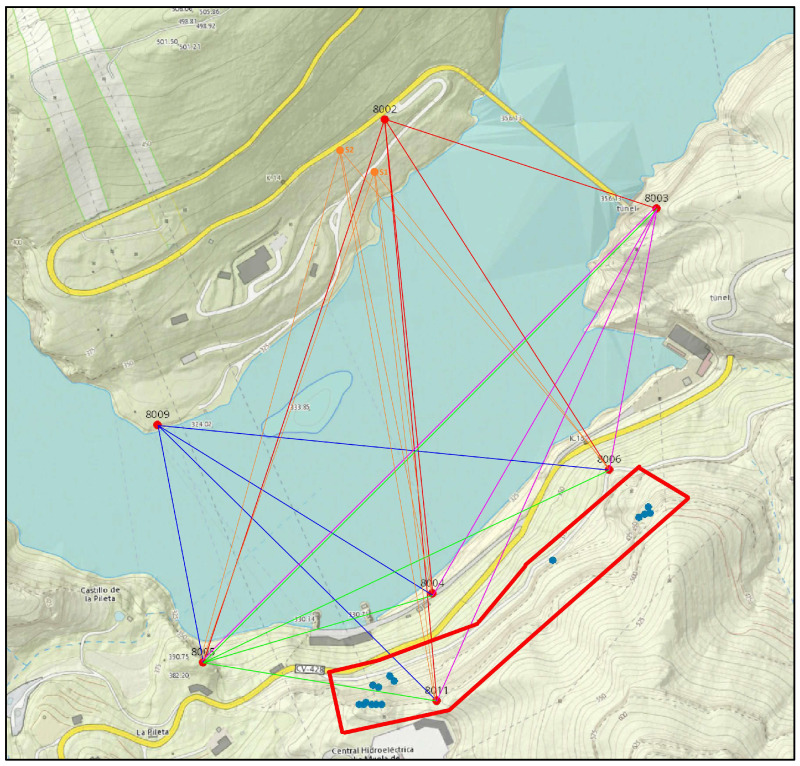
The study area of Cortes de Pallás and the placement of network points. GigaPan stations on pillars 8002, 8003, 8005, and 8009, CPs on pillars 8003, 8004, 8005, 8006, and 8011, ChPs (blue dots without numbering). Observations from each station are shown with different colored lines.

**Figure 2:**
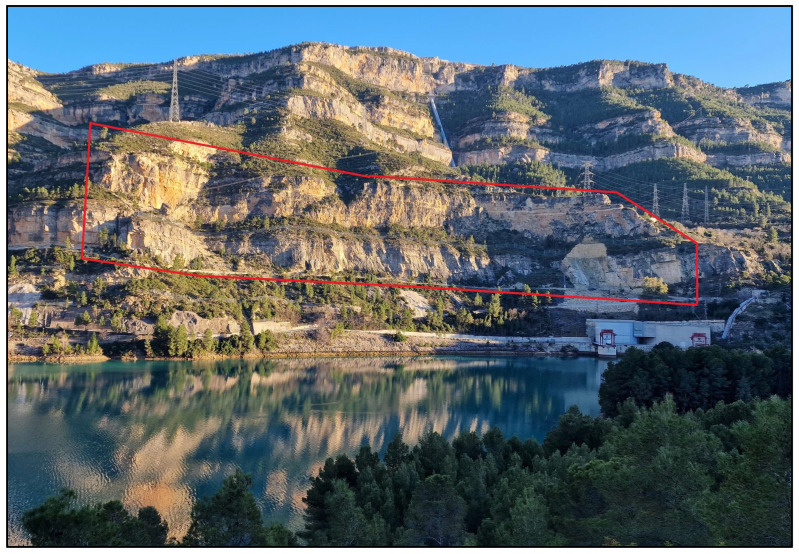
Front view of the rock wall and suspected landslide area.

The critical area in La Muela is a cliff that partially collapsed and seriously damaged the main road and some facilities of the nearby electricity power plant in 2015. In particular, the area of interest is a steep wall facing a water reservoir surrounded by complex orography, which involves measuring distances ranging from 500 m to 2000 m with height differences nearly reaching 500 m (Figure 1 and Figure 2). Furthermore, the presence of a small island with dense vegetation between the area of interest and some stations deprives the full view from the opposite shoreline and prevents the measurements from having good geometry [5]. Taking into account this geometrical and physical limitation, the geodetic network was carefully designed to serve as a precise reference frame so that sub-millimetric distance measurements were optimally carried out to detect possible general instabilities in the area and also to facilitate the proper integration of measurements performed by using other geomatics techniques. However, when it comes to the massive capture of information not limited to several tens of well-defined discrete points, the different technical solutions still find particular problems and limitations depending on their technical nature.

This complex is also a good area for the proper integration of different geomatics solutions to tackle the diverse aspects that many long-term deformation projects usually entail. Particularly in this case, the detection of possible displacements of a few centimetres with the required level of significance in a short period, e.g., several years, becomes a challenging task that requires the joint use of the three aforementioned components, which have to be rigorously integrated [9,10,25,26].

The first component, i.e., the reference frame in La Muela, is realized by means of a geodetic network of ten geodetic pillars, which was monitored from the year 2018 to the year 2020 by using sub-millimetric EDM techniques, dedicated reflectors, and meteorological data loggers on each pillar used to eliminate atmospheric refraction. With such high accuracy, instabilities of several millimetres can be significantly detected and, therefore, the coordinates of pillars can be safely used as known coordinates [5,6].

Once such a high-accuracy geodetic reference frame is available, the techniques used to collect massive information such as TLS, traditional TPh, or PPh, as proposed in this experiment, must be optimally integrated into it. In this study, seven of the ten pillars were used for the proper integration of PPh. Pillars 8002, 8003, 8005, and 8009 were used as stations for the GigaPan device, while pillars 8003, 8004, 8005, and 8006 were used to set up ∅500 mm target spheres when serving as CPs for absolute orientation. In addition, an auxiliary pillar with similar physical features (8011) was included to reinforce the geometry for registration purposes. According to the geodetic adjustments, the accuracy of this auxiliary pillar is slightly lower than those forming the original frame; however, being estimated better than 1 cm for their three components, it can be safely used as part of the reference frame.

The second component, i.e., reliable detection in a short period of possible displacements of a discrete number of relevant points, was carried out by using the same sub-millimetric EDM technique as in the case of the reference frame. However, limiting factors such as the use of 360° reflectors, target points without meteorological information, or weak geometry diminish the accuracy of the coordinates obtained for this type of point by one order of magnitude in comparison with those belonging to the reference frame, i.e., from 1–3 mm to around 1 cm. Fifteen points of this type were used as check points (ChPs) to evaluate the proposed method.

Finally, the third component, i.e., the fast collection of massive data, was required with a frequency higher than once a year or just after relevant events like strong rains or micro-earthquakes. The area of this particular component is limited to 500 m long and 120 m wide (Figure 1 and Figure 2), and the required accuracy is considered to be better than 10 cm. Among the possible techniques able to generate dense 3D models with an overall accuracy better than 10 cm are TLS and image-based techniques. In particular, the present research evaluates PPh as a potential contribution to this third component.

## 3. Study Design and Methods

The research conducted in this experiment takes the following into account: (a) CPs and ChPs provided by the geodetic surveying with well-known coordinates, (b) the 3D point clouds provided by TLS, and (c) the coordinates provided by the proposed PPh for singular point determination as well as for 3D point clouds.

### 3.1. Control Points and Check Points

The CP and ChP coordinates used as true values in this study were obtained by periodical geodetic monitoring from the year 2018 to the year 2020. The method used distances measured with a dedicated sub-millimetric EDM Kern Mekometer ME5000 (Aarau, Switzerland), a network of meteorological sensors, and all the metrological requirements to provide coordinates with millimetric accuracy so that the reliable detection of possible displacements could be performed in a short time [5,6]. The coordinates were determined in the ETRS89 geodetic reference system and subsequently transformed into a more convenient local system denoted as CP2017 [5].

**Figure 3:**
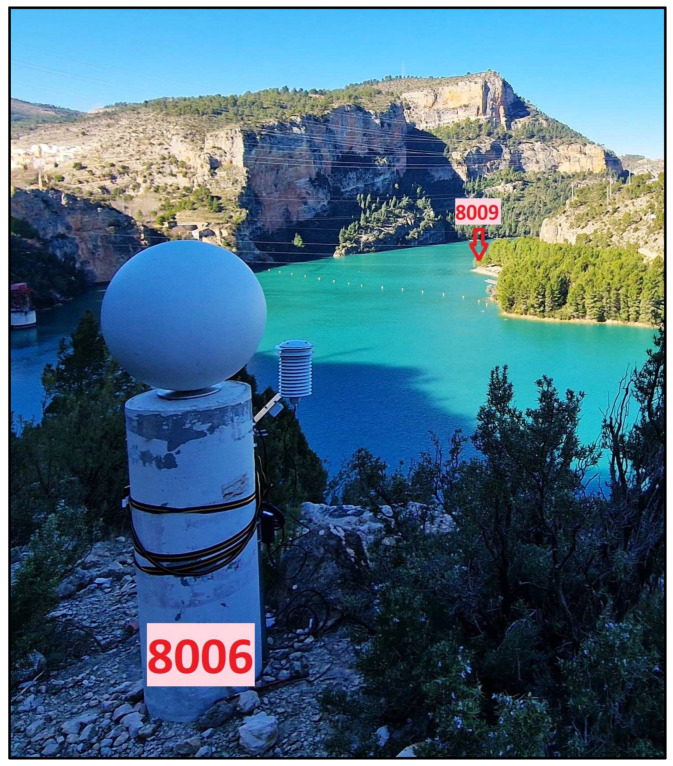
Pillar 8006 is equipped with a ∅0.5 m target sphere and a meteorological data logger used to eliminate the refraction error. Pillar 8009 is marked in red.

Although CP and ChP coordinates were determined by using the same geodetic method, their final accuracy must be considered differently. CPs are materialized by robust geodetic pillars made of concrete and equipped with centring systems on their tops, whose coordinates are known with an accuracy better than 1 mm and 3 mm in the horizontal and vertical components, respectively. These CPs are used in this study either as stations for the GigaPan device (Portland, OR, USA) or as target points for absolute orientation purposes. When used as target points, pillars are equipped with target spheres (∅500 mm) that proved valid to be measured by using both TLS and PPh techniques (Figure 3). The final accuracy of CP coordinates is diminished in comparison with that corresponding to the top of pillars because of uncertainties in the determination of centres of both the GigaPan device and target spheres. In all cases, the contribution of the uncertainty in centre determination is assumed to be lower than 5 mm. Thus, the overall accuracy for CPs can be safely estimated to be around 1 cm, which is clearly better than the expected precision for TLS and PPh.

**Figure 4:**
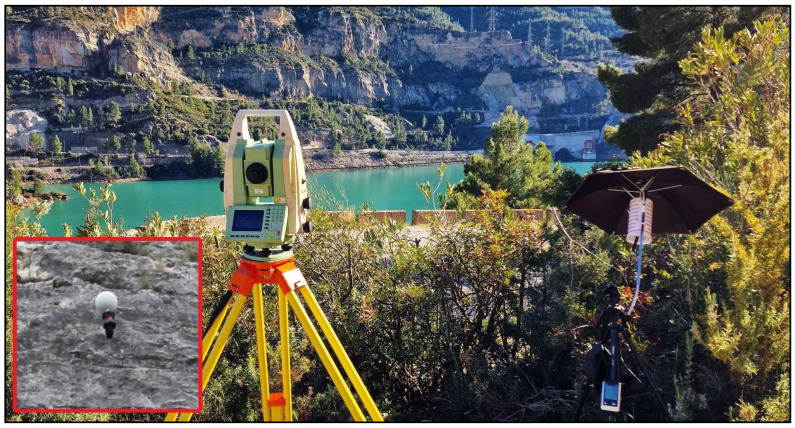
Robotic TS for automatic observation of ChPs during image acquisition. Framed in red is an example of the target installed at the ChPs.

On the other hand, the 15 ChPs are materialized by a set composed of a white sphere (∅145 mm) installed on top of a 360° prism (Figure 4). This set is rigidly anchored onto the solid rock facade. This configuration ensures enhanced visibility and precise target localization for both EDM and image-based techniques [5]. The coordinates of this type of point are assumed to have a precision of around 1 cm because of the operational differences. However, their coordinates must be considered in this study with lower reliability in comparison with the more accurate centring systems on top of pillars because small rockfalls may have displaced the ChPs across the geodetic campaign. Thus, to be on the safe side, a robotic total station, Leica TM30 (Heerbrugg, Switzerland), continuously measured the ChPs during the whole process of image acquisition [5,27,28]. A network of meteorological data loggers (air temperature, air pressure, and humidity) was used to eliminate the refraction error from the TS distances and vertical angles. Since the coordinates obtained by using the TS simultaneously to the image collection proved compatible (only one reflector seemed to have been displaced) with those obtained in the last campaign with the sub-millimetric Mekometer ME5000, the latter, along with the last spatial orientation to propagate them to the centre of the target spheres, were safely retained as known coordinates of ChPs for subsequent comparisons.

The ground-truth coordinates used in this study are shown in Table 1.

### 3.2. TLS Data Acquisition and Registered Point Cloud

**Figure 5:**
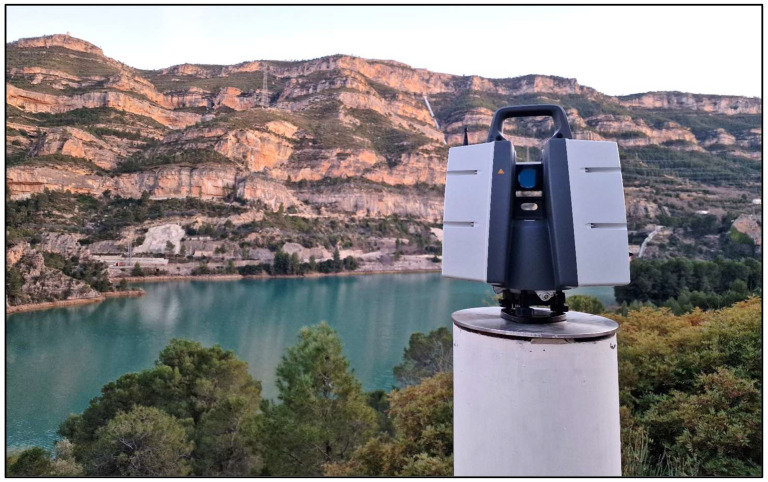
Leica P50 Terrestrial Laser Scanner set up on pillar 8002.

As the basis for comparison with the point cloud obtained from PPh, TLS data play a pivotal role in this study [6,22,29]. A Leica ScanStation P50 was used to collect point clouds from selected pillars of the geodetic reference frame (pillars 8002 and 8009) (Figure 5).

**Figure 6:**
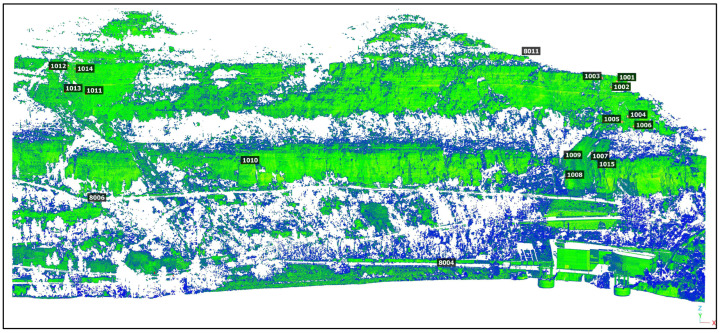
TLS-registered point cloud of the monitoring area.

To prevent errors resulting from merging different point clouds, the TLS point cloud used for comparing with PPh was obtained only with data from pillar 8009, covering the entire monitoring area (Figure 6).

After removing the extra area and filtering the noise points, the georeferencing process to transform the point cloud to local system CP2017 was carried out as follows. First, the coordinates of the centres of the ∅50 cm target spheres were determined. Since the Leica ScanStation P50 has only 60% reflectivity at ranges above 500 m, the average number of points reflected back from the ∅50 cm target spheres was easily lower than several tens or even less. This prevented the use of automatic target recognition, so an assisted process had to be applied by adjusting a ∅50 cm sphere to those points that were manually selected.

Secondly, to maintain geometric consistency and ensure that the known coordinates of the station were preserved, in-house software was used to apply a Helmert transformation with six parameters (translations and rotations) without including any scale factor.

The overall quality of the transformation applied can be evaluated in terms of the residuals obtained for the coordinates of the centre of the target spheres. The average residual obtained was around 1.4 cm.

The range and angular accuracy of this instrument are 3 mm + 10 ppm and 8″, respectively, and the nominal accuracy at distances of around 0.6 km is expected to be around 2 cm. However, taking into account that this instrumental accuracy is normally diminished by additional sources of error, e.g., atmospheric refraction, geometric calibration, or registration issues, the expected accuracy for coordinates for each single point in the cloud is assumed to be around 2–3 cm or even worse depending on the geometrical quality of the registration process [30].

One problem for the TLS technique is the measurement of the ChPs since their small ∅14.5 cm spheres at 600 m could not be detected. On the contrary, when using PPh, those spheres were detected clearly, and their coordinates were individually determined. Thus, the ChP coordinates obtained by using geodetic techniques can only be used for the validation of image-based methods. Consequently, only overall comparisons between the two types of 3D point clouds, i.e., the TLS ones and the one generated by PPh, were carried out.

### 3.3. Panorama Photogrammetry External Orientation

**Figure 7:**
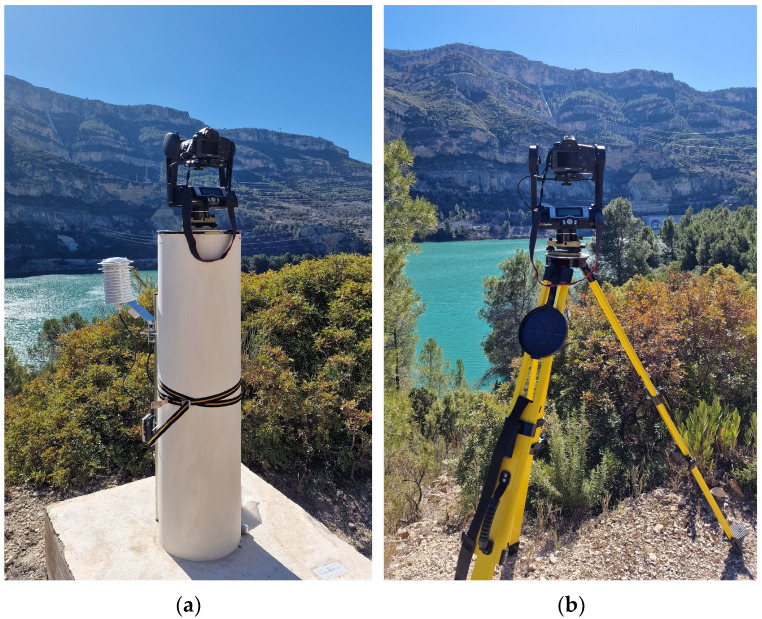
(**a**) Camera with a 200 mm focal length on GigaPan, on a pillar as a main station. (**b**) Camera with 105 mm focal length on GigaPan, on a tripod as an additional station.

The camera used was a digital Canon EOS 5DS R (Tokyo, Japan) full-frame with two lenses including a Canon 200 mm prime lens for finer details and an additional Canon 70–105 mm zoom lens to cover the whole targeting area. The GigaPan device, as a rotation robotic head, was used for the sequential acquisition of overlapped (30–80%) images. The camera was mounted on the GigaPan device and equipped either on a pillar or on a tripod using a tribrach (Figure 7). Since accurate panoramic images require the robotic head to rotate around the camera’s nodal point to eliminate parallax [31,32], the nodal point for the lenses used in the experiment was previously determined in the laboratory and subsequently taken into account when setting up the GigaPan device during the fieldwork [24,32,33].

The fieldwork faced complex lighting conditions and obstructions like vegetation, high-voltage power towers, and a water reservoir island. To overcome these challenges, some initial shots were taken to adjust the exposure of the camera to obtain the right white balance. Then, to reduce visual obstacles, the GigaPan range was adjusted based on each station’s specific view, and stations were selected at different heights and on the opposite line of the target area.

Concerning the experiment design, this method had to fulfil several requirements. First, the set of images captured from each station had to include all the CPs in the front (red dots in Figure 1) for the external orientation and the 15 ChPs (blue dots in Figure 1) for assessment purposes. Second, the number of selected stations had to be optimized with the following two key points in mind: (1) the number of stations had to be as minimum as possible so that the total time for taking images could be associated with a unique observing epoch and (2) the station’s network had to provide accurate 3D models.

To acquire panoramic images, a selection of fixed pillars, including 8002, 8003, 8005, and 8009, was selected as photography stations with a 200 mm lens (red dots in Figure 1). Additionally, 8003, 8004, 8005, 8006, and 8011 were designated as CPs (Figure 3); 8003 and 8005 were used as stations and CPs; and ChPs 1001 to 1015 were placed on the rocky wall (blue dots in Figure 1). Two additional free stations, S1 and S2 (Figure 7b), were used with the 105 mm lens to ensure broader coverage. These two stations were selected according to the conditions of the area and accessibility for stationing, as well as proper visibility in front of the target area (orange dots, Figure 1).

Although the selected values for forward and lateral overlapping was 60% on average, computation parameters such as Ground Sampling Distance (GSD), Horizontal Field of View (H-FOV), Vertical Field of View (V-FOV), and the number of images per station (panoramic acquisition time) were different. Considering the average distance of each photography station to the target area, the GSD values for stations 8002, 8003, 8005, and 8009 were 2.02, 1.66, 1.19 and 1.58 cm/px, respectively, and for stations S1 and S2, 3.52 and 3.72 cm/px, respectively. On the whole, 466 images were taken. By using the 200 mm lens, 88 images were acquired from station 8002 (4 × 22), 66 images from station 8003 (6 × 11), 176 images from station 8005 (11 × 16), and 108 images from station 8009 (6 × 18); for both S1 and S2 stations, 14 images (2 × 7) were acquired.

**Figure 8:**
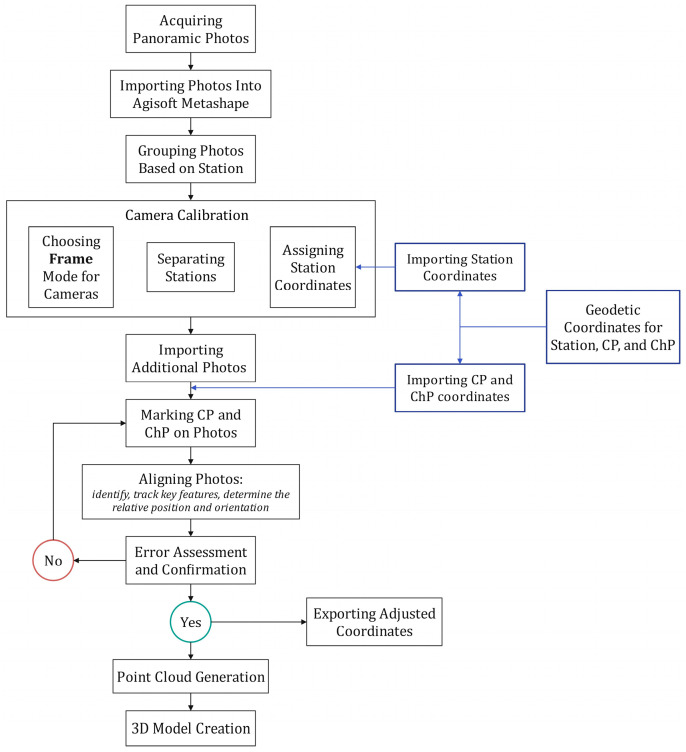
The PPh method workflow.

**Figure 9:**
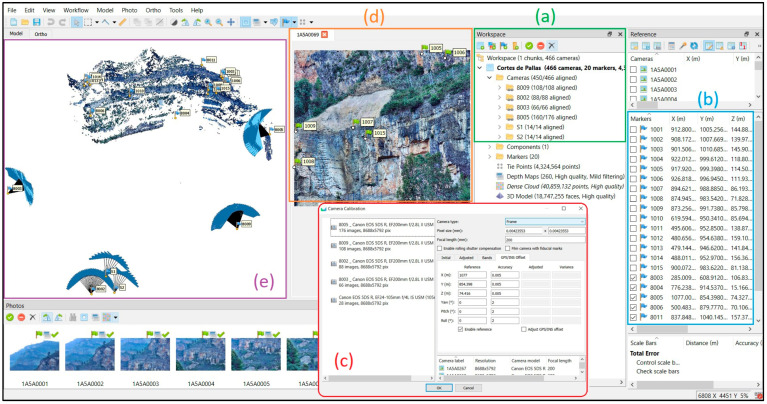
Importing photos and information into Agisoft Metashape and specifying processing parameters.

Image processing and alignment were carried out using Agisoft Metashape software version 1.8.3. The processing steps went according to the software’s workflow (Figure 8 and Figure 9).

Figure 9 shows the overview from the Agisoft Metashape workspace and different sections during the exterior orientation including the following: (a) grouping all station folders and setting the group type as stations for main pillars; (b) importing the coordinates of CPs and ChPs; (c) setting the camera calibration per station, choosing the frame option; (d) manually marking all points on all images; and (e) sparse point cloud with the locations of stations and CPs and ChPs.

## 4. Results and Discussion

### 4.1. Analysis of Coordinates

Table 2 shows the difference between the coordinates obtained by panorama processing and the true value as provided by the geodetic methods. The points in Table 1 are classified into CP, ChP, and stations, documented by geodetic surveying coordinates for each point. The “Panorama Coordinates” in this table denote the adjusted and final coordinates for each point calculated by Agisoft Metashape. Moreover, the disparity between the two coordinate sets is presented, and the Root Mean Square Error (RMSE) values are individually computed for each class.

Considering that the average distance between the fixed pillars and the study area is about 600 m, the distance between stations 8002 and 8003 is 376 m, the distance between 8002 and 8009 is 493 m, and the distance between 8009 and 8005 is 321 m. So based on this, the Distance-to-Base ratio is 1.58, 1.17, and 1.80, respectively. In the following analysis, point 1013 in the series of ChPs was removed from the list of points because of inconsistency in processing, and finally, the project was completed with five CPs and 14 ChPs.

The RMSE of the CPs indicates that the residuals of the transformation were 2.1 cm on average. On the other hand, the RMSE of the ChPs was 1.8 cm. Notably, the dimensions of the ChP spheres play a significant role in determining the achievable precision. Smaller, well-defined spheres are generally conducive to higher accuracy outcomes, whereas larger objects may introduce uncertainties. Moreover, the manual marking of ChP centres across all images introduces a potential source of error. Despite efforts to ensure consistency, minor discrepancies in marking could influence the final photogrammetric outputs. Optimizing sphere dimensions and refining marking procedures could further enhance the method’s precision in monitoring target points.

Finally, the discrepancies found in the coordinates of stations were less than 10 cm. This difference can be partially explained by factors such as atmospheric refraction variation from different stations and related minor errors such as measurement of the height of the GigaPan device and mechanical vibrations during the operation. As expected, modification of the positioning weights in the adjustment did not affect as the final results of the external orientation. It is worth noting that stations S1 and S2 were excluded from Table 1 and Table 2 and added as free stations solely for coverage purposes.

### 4.2. Point Cloud Analysis

**Figure 10:**
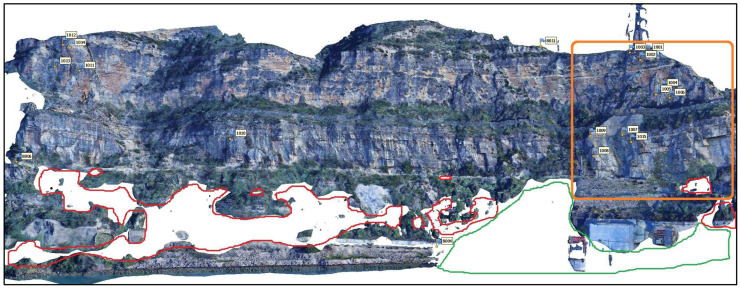
PPh-generated 3D model of the entire study area from the six stations (Figure 9e). The selected area for the point cloud study is framed in orange.

**Figure 11:**
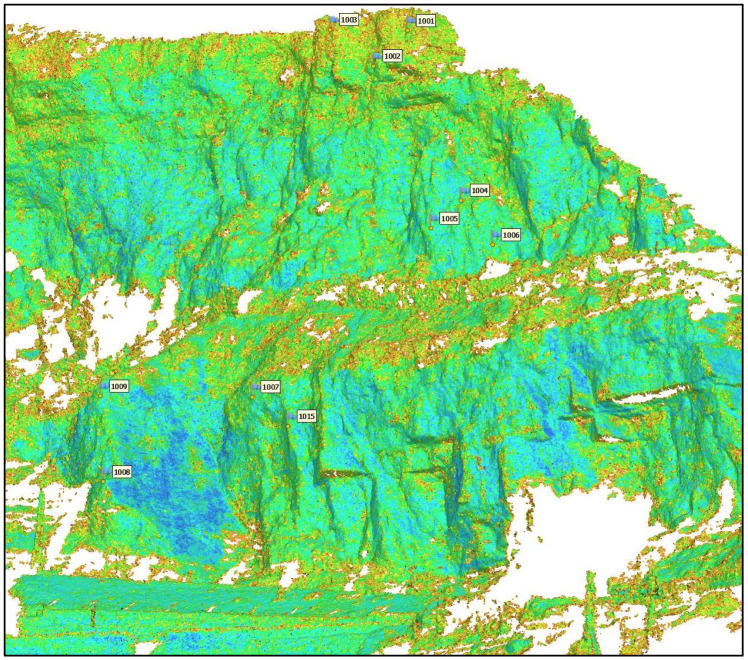
Dense point cloud generated by PPh for the selected area in orange (Figure 10).

Once the accuracy of the ChP coordinates and the external orientation of the proposed panorama method was confirmed, the point cloud and mesh were generated. Figure 10 shows an overview of the generated 3D model of the study area. For the sake of reliability and efficiency, the verification of the 3D model was performed in a well-controlled area, which is shown in Figure 10 and Figure 11.

**Figure 12:**
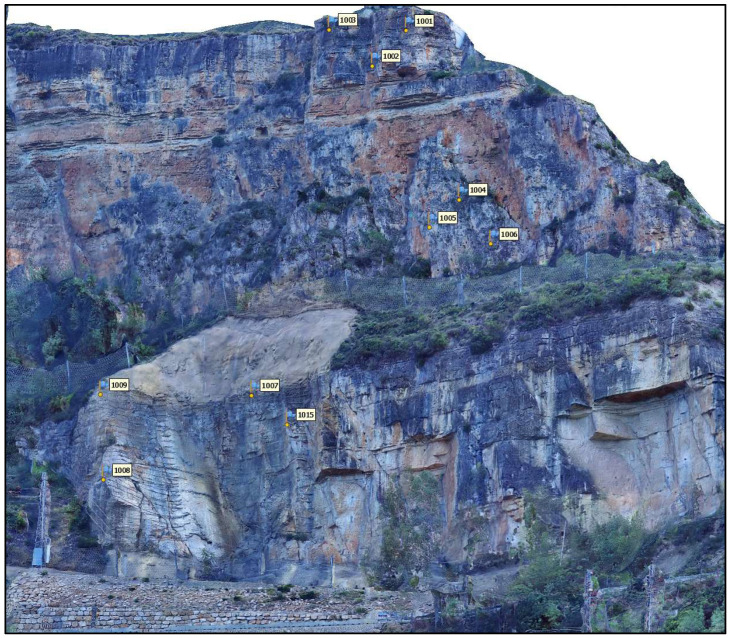
PPh 3D model of the selected area with texture.

Because of the unique and intricate conditions, where vegetation is densely intertwined and tall trees populate the area, the absence of the red border in Figure 11 is related to the dense vegetation within the study area. Additionally, the absence of a green border is due to the presence of trees obstructing the direct sight from the photography station (Figure 10). In the modelling phase, a depth map was used to achieve maximum quality. All the areas suspected of sliding were correctly processed. Figure 12 shows the resulting 3D model of the cropped area after texturing an image of 10,000 × 10,000 px.

**Figure 13:**
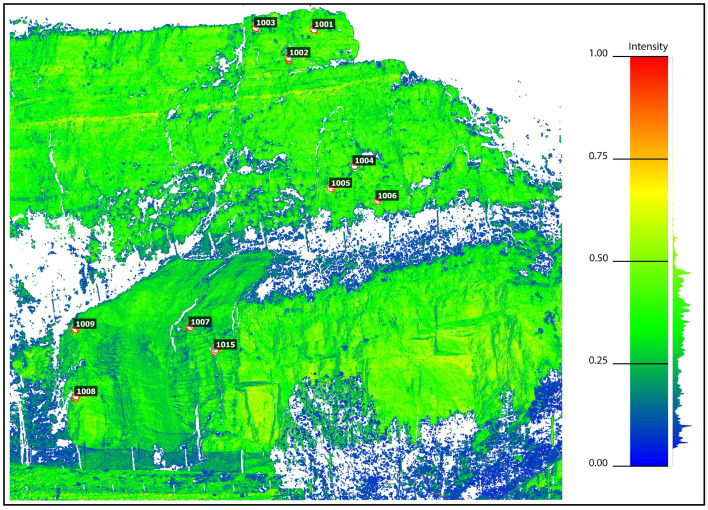
TLS point cloud for the selected area with colour-coded intensity values.

The TLS 3D point cloud data were imported into CloudCompare open-source software, version 2.13, which is displayed with colour-coded intensity (Figure 13). The comparison of two point clouds, TLS and PPh, was also carried out with CloudCompare.

**Figure 14:**
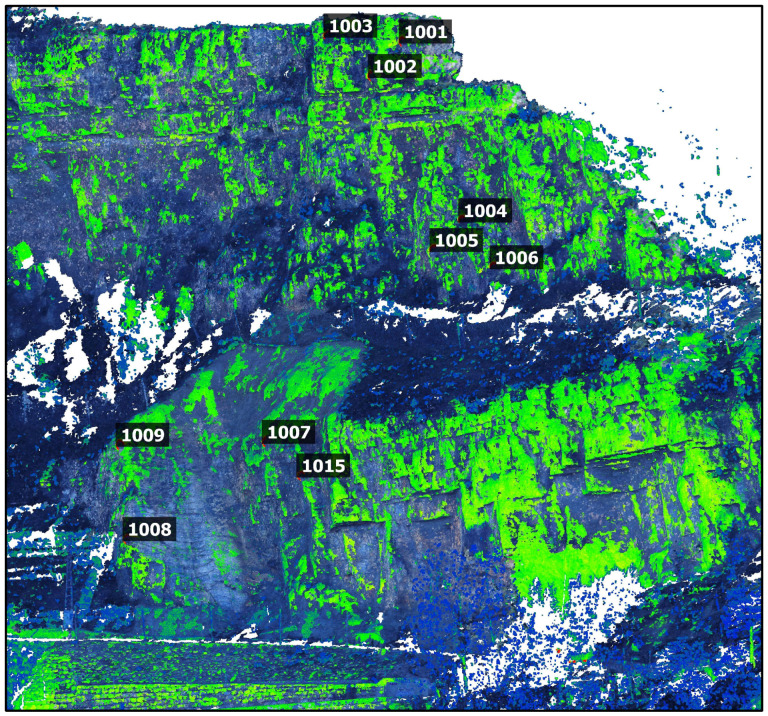
Matching the point clouds of PPh (RGB values) and TLS (colour-coded intensity values).

The cloud-to-cloud distance (C2C) tool in CloudCompare software was used to compare point clouds from TLS and PPh in a selected area (Figure 14) [34]. The tool calculates the Euclidean distance between each point of the compared cloud and the nearest point of the reference cloud. The TLS point cloud was selected as a reference, and the maximum distance was set at 0.5 m.

**Figure 15:**
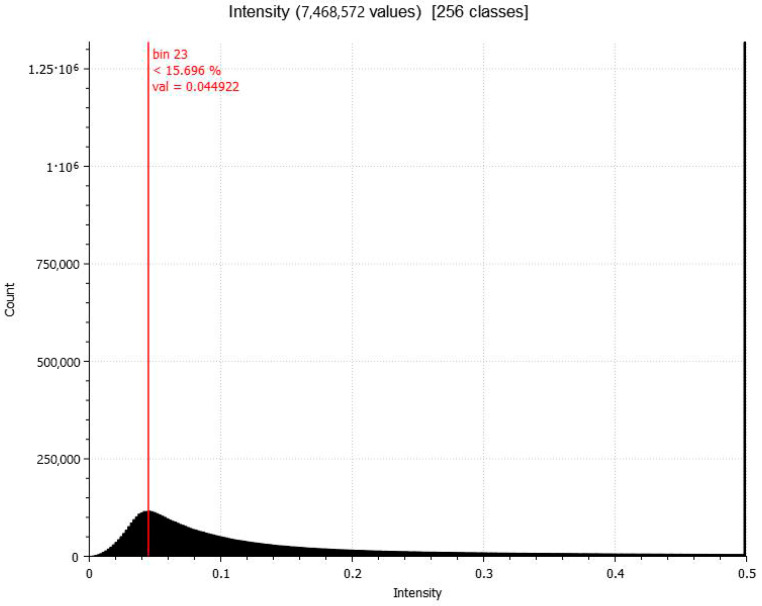
Histogram of the absolute distance value between PPh and TLS point clouds.

The difference between the PPh and the TLS point clouds in the selected area (~6 hectares) is 4.4 cm on average (Figure 15), which is an acceptable rate of compliance compared to TLS at distances above 0.5 km.

PPh ensures continuous evaluation without compromising the structural integrity of the observations in the segments of the main monitoring areas where the target points were located. Considering the region’s topographical complexity, increasing the number of photography stations at various distances and heights provided more comprehensive coverage and a more effective solution for an accurate model. By increasing the number of CPs, one can enhance confidence in accurate georeferencing and scaling. However, PPh facilitates achieving better external orientation even with fewer CPs in the scene. This is because with a wide field of view and capturing a large portion of the scene, sufficient visual information is gathered for orientation and reconstruction.

While PPh offers several advantages, several limitations must be acknowledged. The accuracy of the technique can be affected by factors such as the calibration conditions of the devices, optical image variations especially in long-range monitoring, the presence of reflective surfaces, and, last but not least, the geometry of the image network. To achieve higher accuracy and optimal efficiency, this method must be run within a well-controlled geodetic network with accurate input coordinates. Complications such as obstacles obstructing direct vision or identical and moving textures, like vegetation, may lead to defects or gaps in the point cloud and the delivered 3D model.

## 5. Conclusions

Rigorous deformation monitoring based on periodical measurements requires all measuring campaigns to be integrated into the same reference frame. This mathematical requirement is particularly difficult in vast and inaccessible areas where measurement ranges are longer than 0.5 km. Under these conditions, the integration of TLS or TPh data into a unique reference frame is normally affected by the lack of accurate and well-defined CPs, and the registration process cannot be optimally performed. The usage of PPh proposed in this paper, by combining direct and indirect external orientation, was demonstrated to be a potential approach to extend the adoption of TPh for long-range monitoring as a low-cost technique to overcome TLS limitations.

Concerning the accuracy verification in well-controlled singular points, the results obtained for the 15 ChPs showed that the difference in coordinates regarding those obtained by using geodetic techniques was on average 1.1 cm, 1.1 cm, and 0.8 cm for local coordinates x, y, and z, respectively. It is important to stress that this high accuracy at distances longer than 0.5 km was obtained with targets consisting of small spheres (∅14.5 cm) that would not have even been sensed by TLS. Moreover, the technique detected a possible rotation in ChP 1010, whose x and z coordinates presented displacements clearly above 1 cm.

Concerning point clouds, the accuracy of the proposed method was analysed in two ways. Firstly, considering the quality of the external orientation, and secondly, by statistical analysis of the cloud-to-cloud distance between the PPh-based and the TLS-based point clouds in a selected well-controlled area.

The cloud-to-cloud comparison showed that 15.6% of the points were closer than 4.4 cm. Taking into account that the distance between the analysed point clouds includes a range of sources of error such as registration errors, noise introduced by vegetation and not well-defined natural objects, atmospheric refraction, and modelling, it can be concluded that the proposed technique has a similar appreciation as the nominal one claimed by the TLS method, which is assumed to be around 2–3 cm at the case at hand.

The proposed method still shows several limitations. As pointed out, these limitations include the technique requiring careful camera settings and calibration, the final accuracy strongly depending on the image network, the optical variations during data acquisition, lighting conditions, the panorama device employed, surface albedo, and possible obstructions. Thus, it may well be that the geodetic network, normally designed for the use of EDMs, would not suit the geometrical requirements of PPh. In those cases, additional GigaPan stations should be included and their coordinates carefully obtained from the geodetic network by using geodetic techniques, which in turn would increase the workload and cost of the work field. On the whole, since the cost-effectiveness of PPh is significantly lower than other widely used solutions used for massive deformation monitoring, i.e., TLS, PPh is presented as an attractive alternative approach for long-term monitoring projects with limited budgets.

## Figures and Tables

**Table 1:** Cartesian coordinates expressed in the CP2017 local system.

		CP2017 Local Coordinates		
	Pt. Num	x	y	z
**ChP**	1001	912.800	1005.256	144.885
	1002	908.172	1007.669	139.972
	1003	901.506	1010.685	145.908
	1004	922.012	999.612	118.809
	1005	917.920	999.398	114.500
	1006	926.818	996.945	111.935
	1007	894.621	988.885	86.193
	1008	874.945	983.542	71.828
	1009	873.256	991.738	85.798
	1010	619.594	950.341	85.694
	1011	495.606	952.850	138.870
	1012	480.656	954.638	159.100
	1014	488.011	952.970	156.362
	1015	900.072	983.622	81.138
**CP**	8003	285.009	608.912	106.830
	8004	776.238	914.537	15.166
	8005	1077.003	854.398	74.327
	8006	500.483	879.777	70.106
	8011	837.848	1040.145	157.377
**Station**	8002	536.260	341.249	47.028
	8003	285.009	608.912	106.915
	8005	1077.003	854.398	74.416
	8009	981.742	554.051	10.777

**Table 2:** Coordinates obtained by the PPh method and their correction concerning those obtained from geodetic techniques.

		Panorama Coordinates				Corrections			
	Pt. Num	x	y	z		Cx	Cy	Cz	RMSE Total
**ChP**	1001	912.798	1005.252	144.885		0.002	0.004	0.000	0.004
	1002	908.168	1007.667	139.978		0.004	0.002	−0.006	0.008
	1003	901.499	1010.682	145.912		0.007	0.004	−0.004	0.009
	1004	921.990	999.610	118.807		0.022	0.002	0.001	0.022
	1005	917.920	999.387	114.499		−0.001	0.011	0.001	0.011
	1006	926.811	996.941	111.935		0.007	0.003	0.000	0.007
	1007	894.620	988.880	86.188		0.001	0.005	0.005	0.007
	1008	874.938	983.528	71.827		0.007	0.014	0.001	0.016
	1009	873.249	991.723	85.794		0.007	0.015	0.004	0.017
	1010	619.617	950.337	85.681		−0.023	0.004	0.013	0.027
	1011	495.592	952.866	138.866		0.014	−0.016	0.004	0.021
	1012	480.652	954.663	159.111		0.004	−0.025	−0.011	0.028
	1014	487.998	952.976	156.369		0.013	−0.005	−0.007	0.016
	1015	900.056	983.610	81.119		0.016	0.013	0.019	0.028
					RMSE	0.011	0.011	0.008	0.018
**CP**	8003	285.023	608.912	106.836		−0.015	−0.001	−0.006	0.016
	8004	776.230	914.526	15.148		0.008	0.010	0.018	0.022
	8005	1077.009	854.399	74.318		−0.007	−0.001	0.009	0.011
	8006	500.490	879.777	70.104		−0.007	−0.001	0.002	0.008
	8011	837.848	1040.159	157.409		0.001	−0.014	−0.032	0.035
					RMSE	0.009	0.008	0.017	0.021
**Station**	8002	536.150	341.295	47.082		0.110	−0.046	−0.054	0.131
	8003	284.902	608.890	106.941		0.106	0.022	−0.026	0.112
	8005	1076.946	854.405	74.459		0.056	−0.007	−0.043	0.071
	8009	981.755	554.035	10.751		−0.013	0.016	0.026	0.034
					RMSE	0.082	0.027	0.039	0.095

## Data Availability

Data are contained within the article.
